# P52 Survey on the experience of children and families attending the Paediatric Rheumatology daycare unit during the COVID pandemic

**DOI:** 10.1093/rap/rkac067.052

**Published:** 2022-09-28

**Authors:** Pirathini Mohan, Muhammad Zulkifli, Rebecca Ward, Sam Deepak, Kishore Warrier, Satyapal Rangaraj

**Affiliations:** The University of Nottingham, Nottingham, United Kingdom; Nottingham Children's Hospital, Nottingham, United Kingdom; Nottingham Children's Hospital, Nottingham, United Kingdom; Nottingham Children's Hospital, Nottingham, United Kingdom; Nottingham Children's Hospital, Nottingham, United Kingdom; Nottingham Children's Hospital, Nottingham, United Kingdom

## Abstract

**Introduction/Background:**

Patients attend rheumatology daycare either for regular infusions or one episode depending on their treatment. A typical day for a child would involve observations, having a cannula put in and having the infusion before returning home. Due to the COVID-19 pandemic, there had been some changes to the day care pathway in our hospital including: only one parent being allowed to attend with their child; a strict timing schedule; COVID-19 testing prior to attending appointments. We felt it is vital to know the young person and the family’s perspectives of their experience at day care.

**Description/Method:**

A special study module (SSM) medical student conducted a feedback survey over a 2 month period (December 2021 and January 2022). The aims and objectives were focused on the following:

- Whether the patients are receiving an appropriate level of care to optimise their experience at day-care.
- was their treatment given in a timely manner?
- Were there any delays, if so the reasons and actions taken to mitigate.

2 questionnaires were used, one for the patient and one for the parent/carers. The questionnaires were provided to the patient/carers during their admission by the admitting nursing staff. The medical student collected the questionnaires at the end of the day.

**Discussion/Results:**

39 patients attended the daycare during this period. 9 completed questionnaires were received.

• Feedback from parents/carers:

- Carers (100%) said they were happy with how they had appointment dates and most appointments took their availability and preferences into consideration. Due to COVID, families were notified of their appointment time only one day prior; this was highlighted as an area for improvement to ensure enough notice for the appointment time.

- All parents (100%) agreed that they received enough information regarding their child’s appointment at daycare and this was delivered in an understandable manner.

- 77.8% (7 out of 9) of parents did not experience long waiting times at their appointment. 22.2% (2 out of 9) parents said there were long waiting times at their appointment, and they were made aware of the reasons regarding the wait. The main reasons highlighted was busy ward with lots of patients.

- 2 out of 7 parents indicate the care provided has remained the same pre- and post-pandemic. 3 out of 7 patients felt that their care has improved due to the following reason: treatment was completed more quickly; wards looked less busy as there were limited patients present at one time; there was a reduced length of stay compared to pre-COVID.

• Children’s questionnaires:

- 100% (9 out of 9) children were happy with the care received at daycare.

- 22.2% (2 out of 9) children reported being worried/anxious due to having bloods and cannula taken (1) and missing school (1).

- 5 out of 9 children had play therapist input and they all found it helpful.

**Key learning points/Conclusion:**

Despite the COVID pandemic restrictions, all young people and families had a positive experience during their day care treatments. Effective communication and engaging the patients and families, and providing appropriate explanations to any delays in treatment is vital. The overall experience has provided an opportunity to reflect on the systems to manage the day care treatments, which we presume has been the case in many centres. Allied health professionals like play therapists and youth teams provide immense support to the young people who are attending day care for regular treatments. Provision of these services to all young people if appropriate will enable positive experiences to the patients. Limitation of this survey was a small sample size with the response rate of only 23%. Further survey over an extended period may provide more insight in this area.

